# Next-generation sequencing yields the complete mitogenome of the Shaw’s sea-snake (Squamata: Elapidae)

**DOI:** 10.1080/23802359.2019.1676667

**Published:** 2019-10-17

**Authors:** Qingbo Qiu, Zhongyin Chen, Zixuan Zhang, Yu Du, Chixian Lin, Xiang Ji

**Affiliations:** Hainan Key Laboratory of Herpetological Research, College of Fisheries and Life Science, Hainan Tropical Ocean University, Sanya 572022, China

**Keywords:** Complete mitochondrial genome, phylogeny, Elapidae, *Hydrophis curtus*

## Abstract

We report the complete mitogenome of *Hydrophis curtus*, which is 17,702 bp in size and includes 13 protein-coding (PCGs), two rRNA genes, 22 tRNA genes, and two control regions. PCGs, with 13 genes, are 11,261 bp in length. All PCGs use as the start codon ATN except ND1 (CTA) and COX1 (GTG); ATP8, ATP6, ND4L, ND5, and Cytb use the typical stop codon TAA; but COX2 and ND4 with a single T-. Phylogeny recon-structed using the Bayesian inference (BI) method with 13 PCGs indicates that *H. curtus* at the root of Laticaudinae.

Shaw’s sea-snake (*Hydrophis curtus*, HYCU), a viviparous species, belongs to the genus *Hydrophis*, and inhabits shallow coastal waters around western Pacific Ocean and Indian Ocean (Zhao and Adler 1993). The genus *Hydrophis* belongs to the family Elapidae and including 48 species (Uetz et al. 2019). The taxonomy of the genus *Hydrophis* has been questioned due to minor morphological variations among species (Zhao and Adler 1993). In order to obtain more basic genetic information for phylogeny on this group of snakes, we determined the complete mitogenome of *H. curtus*.

The sample (HKHR‒HYCU‒20180303) was donated by the local fishermen from the coastal of Hainan island, China, and stored in a freezer (WUF‒300, DAIHAN^®^ Digital Ultra-Low Temp. Freezer, DAIHAN Scientific Co., Ltd., Korea) at ‒80 °C in the Museum of Hainan Key Laboratory of Herpetological Research (HKHR) at Hainan Tropical Ocean University in Sanya, Hainan, China. We chose the muscle tissue to extract the total genomic DNA using EasyPure@ Genomic DNA Kit according to the manufacturer's instructions (TransGen Biotech Co, Beijing, China). The mitogenomes of *H. curtus* were sequenced by next-generation sequencing (Illumina HiSeq X ten), and clean data without sequencing adapters were *de novo* assembled using the NOVO Plasty 2.7.2 software (Dierckxsens et al. 2017). The complete and circular mitochondrial genome is 17,430 bp in size, with an AT bias of 60.0%, and includes 13 PCGs (11,261 bp, ND1‒6, ND4L, CO1‒3, Cytb, ATP6 and ATP8), two rRNA genes (*rrnS* and *rrnL*), and 22 tRNA genes and two non-coding regions (CR or D-loop). All PCGs use as the start codon ATN except ND1 (CTA) and COX1 (GTG); ATP8, ATP6, ND4L, ND5, Cytb use the typical stop codon TAA; but COX2 and ND4 with a single T. Twenty-eight gens are encoded on the H-strand and eight genes (ND6 gene and 7 tRNAs) are located on the L-strand. The 22 tRNA genes varied in size from 57 to 73 bp. There are 14 overlapping regions in the mitogenome with 1–38 bp size. The mitogenome of *H. curtus* has been deposited in Genbank under accession number MK953549.

Using two Viperidae species as outgroups, the phylogeny of 10 Elapidae species including *H*. *curtus* was reconstructed based on 13 PCGs by the Bayesian inference (BI) methods through MrBayes (version 3.2.2). The resultant BI tree distinctly indicated that *H*. *curtus* belongs to a monophyletic group including Elapinae, Laticaudinae and Bungarinae, and *H*. *curtus* is located at the root of the Laticaudinae (Figure 1).

**Figure 1.**
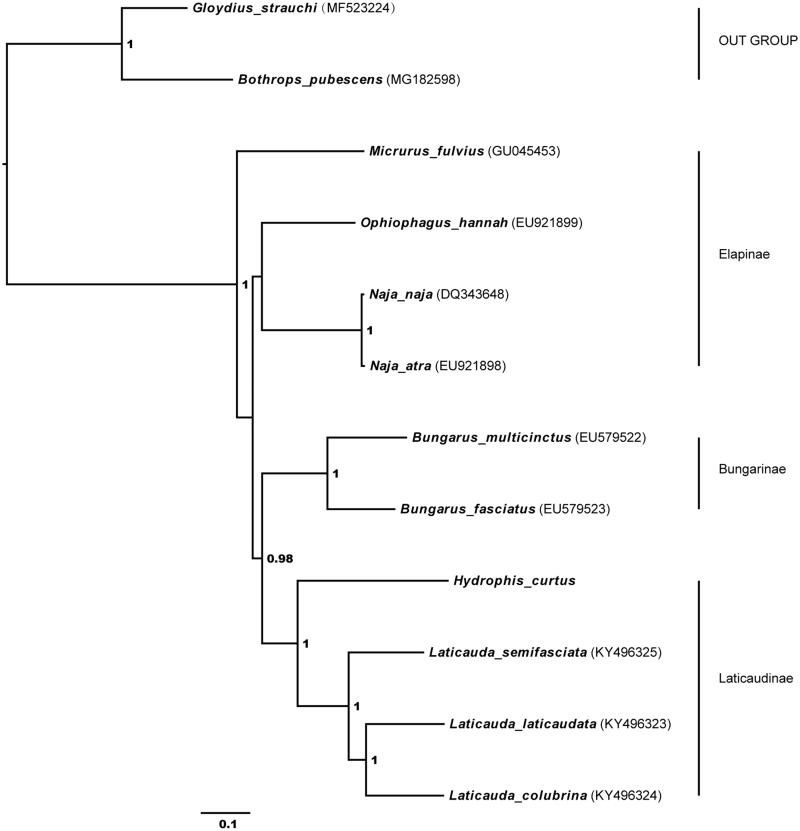
Bayesian inference (BI) phylogenetic tree inferred from the nucleotide sequence data of mitogenomic 13 PCGs.
